# Comparing positive versus negative intrinsic rewards for predicting physical activity habit strength and frequency during a period of high stress

**DOI:** 10.1111/aphw.12650

**Published:** 2025-02-04

**Authors:** Lindsey Fremling, L. Alison Phillips, Lindsay Bottoms, Terun Desai, Katie Newby

**Affiliations:** ^1^ Department of Psychology Iowa State University Ames Iowa USA; ^2^ Centre for Research in Psychology and Sports University of Hertfordshire Hatfield UK; ^3^ Institute of Sport, Exercise and Health, Division of Surgery and Interventional Science University College London London UK

**Keywords:** COVID‐19, habit strength, intrinsic motivation, intrinsic rewards, physical activity, stress reduction

## Abstract

The experience of positive intrinsic rewards (enjoyment) from physical activity (PA) is known to promote PA habit formation and maintenance. Negative intrinsic rewards (stress reduction) may also be associated with PA habit, particularly during a major stressor and when individual‐level anxiety is higher. Multi‐level models tested the following hypotheses using weekly survey data from a convenience sample (snowball sampling) of adults (*N* = 580; 91% White, 77% Female, mean age = 41 years) over the 8 weeks of the first COVID‐19 lockdown in the UK: negative intrinsic rewards will be independently and statistically more strongly related to PA habit strength and frequency than positive intrinsic rewards; and, the relationship between negative intrinsic rewards and PA habit strength and frequency will be stronger for those with higher anxiety. Counter to the hypotheses, positive intrinsic rewards were more strongly associated with PA habit strength over time than negative intrinsic rewards (fixed effect = 0.27, *p* < 0.001 versus fixed effect = −0.05, *p* = 0.23, respectively), and there was a main effect of anxiety (but no interaction with negative rewards) on PA habit strength (fixed effect = −0.03, *p* = 0.03). The findings suggest that interventions aimed at increasing and maintaining PA habit strength might best focus on cultivating positive intrinsic rewards (enjoyment) from PA, even in the presence of substantial stressors and individual‐level anxiety.

## INTRODUCTION

Physical activity (PA) has been shown to not only diminish the risk, and delay the onset of, a wide variety of chronic conditions and diseases but also reduce risk of mortality (Garcia et al., 2023; Ruegsegger & Booth, 2018). In addition to the physiological benefits, PA has also been shown to be beneficial in the treatment of mental and cognitive health conditions (Mahindru et al., 2023; Ruegsegger & Booth, 2018). Despite these well‐documented benefits, average levels of PA are far below that recommended for health, for example, over 35% of UK adults do not engage in sufficient activity levels (NHS England, 2023).

To reap the extensive benefits that PA has to offer, this behavior needs to be both initiated and maintained over the lifetime (Hargreaves, 2021; Sherwood & Jeffery, 2000; Stonerock et al., 2015). The findings of research on PA initiation provide cause for optimism, for example, a meta‐analysis by Gourlan et al. (2016) of theory‐based PA interventions (i.e., those based on recognized theories of behavior and which target the theorized mediators of behavior change) shows that, of the 82 interventions examined, there was a moderate, positive effect on PA. What studies also show, however, is that engaging in regular PA over the long‐term is challenging, with promising early effects often being short‐lived (Arikawa et al., 2012; Farooq et al., 2019; Gabay & Oravitan, 2022; Marcus et al., 2000).

Knowledge about what strategies support PA maintenance is still relatively new (Deslippe et al., 2023; Kwasnicka et al., 2016; Marcus et al., 2000). The development of a PA habit (i.e., automatic enactment of PA in a conditioned context) is a promising way to improve long‐lasting PA engagement (Rhodes & Rebar, 2018), since habits are known to be consistent, frequent, resistant to change, and enacted despite changes in outcome‐related goals (i.e., an exercise habit persists despite changing external goals, such as to lose weight or alter one's appearance) (Gardner, 2015).

One factor that has been shown to promote PA habit formation (Gardner & Lally, 2013) and maintenance is the experience of intrinsic rewards from PA, or intrinsic motivation to engage in PA (Phillips et al., 2016; Phillips & Mullan, 2022). The experience of rewards intrinsic to the behavior (enjoyment) is the source of individuals' intrinsic motivation to engage in that behavior in the future, as delineated by self‐determination theory (SDT)—a comprehensive theory of motivated behavior that originated from the humanistic perspective and is centered around the fulfillment of needs, self‐actualization, and the realization of the human potential (Deci & Ryan, 2000). SDT proposes that this “quality of motivation” will influence the extent to which individuals will engage in and persist with a behavior, with intrinsic motivation (i.e., motivation centered around the attainment of an intrinsic reward) regarded as higher quality than extrinsic motivation (i.e., motivation centered around the attainment of an extrinsic reward). As such, it is theorized that longer term PA maintenance is best promoted through a focus on intrinsic motivation and the intrinsic rewards one can attain through engagement in PA (as opposed to extrinsic motivation and rewards).

Combining SDT principles with theory on habit formation (Gardner & Lally, 2018), intrinsic rewards from PA can promote behavioral repetition in the short‐term (promoting behavioral initiation) via stronger intentions (Phillips et al., 2016), and when behavioral repetition occurs in a stable context, habit formation is facilitated (Gardner & Lally, 2013). Further, as outlined in recent literature (see Phillips & Mullan, 2022), behaviors that are complex, such as physical activity, need rewards for habitual enactment to form and, further, to be maintained—without ongoing reward, habitual behavior in response to a learned cue will dishabituate (see Phillips & Mullan, 2022). Phillips et al. (2016) found that the experience of intrinsic rewards predicted PA frequency among maintainers via their habit strength.

Existing research has primarily operationalized intrinsic behavioral rewards as positive intrinsic rewards (see Teixeira et al., 2012)—rewards that come in the form of a gain in something positive upon behavioral performance, such as enjoyment or satisfaction. In the current study, we build from the recent habit and SDT literature to evaluate the potential role of negative intrinsic rewards on physical activity (PA) habit strength and frequency—negative intrinsic rewards are achieved when PA removes negative stimuli, for example, reducing stress or anxiety. Phillips et al. (2016) is the only study, to our knowledge, that has included both a measure of positive intrinsic reward and negative intrinsic reward in their examination of PA habit strength. However, in their analysis, they combined their measures of negative and positive intrinsic rewards and did not investigate the independent role of negative intrinsic reward.

Existing research suggests that the removal of a negative stressor can serve as a significant motivator for continued PA engagement. For instance, Ednie and Stibor (2017) found that using PA as a form of stress management was a significant predictor of total PA scores. They also included an open‐ended response question to better assess how the various variables of interest either did or did not serve as PA motivators, and results from the coding of these responses suggested that those who attained a reduction in stress through PA experienced it as intrinsic motivation. Research has also found that when moods improve as a result of PA engagement, it is usually due to decreases in tension, depression, anger, and confusion (Berger & Motl, 2000). Additional studies have also been able to demonstrate that acute PA is significantly effective in reducing self‐reported stress levels and stress as measured through blood pressure responses (Crocker & Grozelle, 1991; Ebbesen et al., 1992; Hamer et al., 2006). These findings highlight the important role that negative rewards (e.g., removal or lessening of anxiety or stress) may play in PA engagement. With regard to habit strength in particular, Stults‐Kolehmainen and Sinha (2014) present evidence that individuals with strong PA habits engaged in PA as a means of coping with stress—that is, under stress, those with strong PA habits engaged in more PA, whereas those with weak PA habits engaged in less PA. This research suggests that negative intrinsic rewards, specifically stress reduction with PA, may maintain PA habit strength when under stressful conditions.

Therefore, we propose that, like positive intrinsic rewards, the experience of negative intrinsic rewards from PA may be related to stronger PA habits. Further, this may be particularly the case in the presence of a substantial stressor and individuals' experience of anxiety, since periods of high stress and anxiety would necessitate greater use of one's coping mechanism—PA for those who report negative intrinsic rewards from PA. Conversely, those who experience only positive intrinsic rewards from PA may stop or lessen exercise in times of high stress, instead prioritizing other coping mechanisms and/or perceiving a lack of leisure time to pursue positive intrinsic rewards. Any reduction or disruption in PA would theoretically result in a reduction in PA habit strength over time.

The COVID‐19 pandemic presented itself as an opportune naturalistic context in which to compare the impact that negative and positive intrinsic rewards have on physical activity habits. Past research has established that stress and other negative emotions like anxiety and depression were heightened during the pandemic among UK citizens (Jia et al., 2020; McPherson et al., 2021; Zavlis et al., 2022). Although lockdown was a stressor for all individuals included in the lockdown mandates, there were likely individual differences in stress responses (i.e., level of anxiety) to the stressor of lockdown. Therefore, the current study uses this unique context of COVID‐19 stress, while also accounting for individual reports of experienced anxiety, to fill gaps in our knowledge about the relative importance of negative versus positive intrinsic rewards for exercise habit strength and exercise frequency and to test the following hypotheses:

1. The degree of experiencing both positive and negative intrinsic rewards from PA will be positively associated with subsequent PA habit strength and PA frequency, with experience of negative intrinsic rewards being statistically more strongly related to PA habit strength.
2. The strength of the relationships between negative intrinsic rewards and PA habit strength and between negative intrinsic rewards and PA frequency will be moderated by degree of anxiety reported by the participants, such that the relationships between negative intrinsic rewards and habit strength and frequency will be stronger when anxiety is high versus low.

To test the hypotheses, we examined the constructs over a period of 8 weeks, beginning with the first lockdown in the UK during the COVID‐19 pandemic. To our knowledge, no research to date has directly compared the effects of negative intrinsic reward and positive intrinsic reward on exercise frequency and habit. An examination into how negative and positive intrinsic reward each impact exercise frequency and habit can aide in future exercise intervention programs. Further, research has suggested that pandemics akin to that of the COVID‐19 pandemic will become more frequent in the future (Haileamlak, 2022), necessitating research that assesses physical activity motivators during pandemic settings.

## METHODS

### Participants and procedure

The present study was part of a broader project examining changes in PA levels and habit among UK residents during the COVID‐19 pandemic. Participants included both members of the UK public, recruited via Google Ad and social media (Twitter/X), and staff/students at a UK university, recruited via university‐wide email invitation. Of the 580 consented participants, 22% identified as male, 77% identified as female, and 91% identified as White ethnicity. The mean age was 41 years (SD = 21 years), and 89% reported living with others.

Recruitment took place during the COVID‐19 pandemic, during which time a stay‐at‐home order was in place in the UK with all but essential contact and travel prohibited, and people only allowed to leave the house once per day for PA. Participants had to be aged 18 years or older and living in the UK; those who had been instructed to self‐isolate due to categorization as ‘extremely high risk’ of COVID‐19 or as a shielded patient were not eligible to participate in the study. Baseline and informed consent information were accepted from 14 April 2020 to 20 April 2020, and data collection began on 15 April 2020. Participants provided informed consent at the beginning of the baseline survey; all methods were approved by the corresponding author's institutional review board. While data were collected up until March 2022, only data collected through June 2020 were used in the present study (to capture the time period when the restrictions were newest and likely to be experienced as most stressful and disruptive to individuals' normal PA behaviors). Initially (between 15 April 2020 and 9 May 2020), all participants were asked to complete a daily diary, recording their level, and duration of PA. On Saturdays, these questions were accompanied by additional items measuring the main variables of interest (including positive intrinsic reward, negative intrinsic reward, anxiety, and PA habit). From 3 May 2020 onwards, to reduce participant burden, some participants opted to switch to a weekly version of the diary (*N* = 100 opted to receive weekly surveys; *N* = 480 continued daily PA surveys), received every Saturday and identical to the daily diary except that participants were asked to record PA levels and duration they had engaged in over the past 7 days. Where PA was captured using daily diaries, PA frequency was assessed by calculating an average score over 5, 6, or 7 days of completed daily surveys. If participants returned fewer than 5 diaries in any 1 week, the participant was assigned a missing value for PA frequency that week.

### Measures

There were a variety of different variables examined across the whole research project. The following measures were used to collect data for the variables of interest in the present study.

#### Positive intrinsic reward

Positive intrinsic reward for PA was assessed using a modified version of the Behavioral Regulation in Exercise Questionnaire (BREQ; Mullan et al., 1997) focusing on intrinsic regulation (Cronbach's alpha at baseline = 0.86). Participants were asked to rate their agreement with four statements regarding how they felt about being physically active on a scale of 1 (*strongly disagree*) to 5 (*strongly agree*). The four items were as follows: (1) I do it because it is fun; (2) I enjoy it; (3) I find it pleasurable; (4) I get satisfaction from doing it. Scores across the four items were averaged to create a possible range of scores between 1 and 5.

#### Negative intrinsic reward

Negative intrinsic reward for PA was determined using a measure developed by Phillips et al. (2016) which assesses the degree to which negative reinforcement serves as a motivator for PA. Participants were asked to rate their agreement on two items regarding how they felt about being physically active on a scale of 1 (*strongly disagree*) to 5 (*strongly agree*). The two items were as follows: (1) I do it in order to feel better when I am in a bad mood; (2) I do it in order to remove stress. Scores across the two items were averaged to create a possible range of scores between 1 and 5. Cronbach's alpha at baseline = 0.98.

#### Anxiety

Participants' level of anxiety was measured with the 2‐item Generalized Anxiety Disorder scale (GAD‐2; Kroenke et al., 2007), edited to assess the prior week: “Over the past week, how often have you been bothered by: (1) feeling nervous, anxious, or on edge and (2) not being able to stop or control worrying.” Answer options ranged from not at all bothered (0) to very bothered (6). Responses across the two items were summed to create a total range of scores between 0 and 12.

#### Physical activity habit

Habit was measured using the Self‐Report Behavioral Automaticity Index (SRBAI; Gardner, 2012; Cronbach's alpha = 0.91). Participants were asked to rate their agreement with four items relating to being physically active on a scale of 1 (*strongly disagree*) to 5 (*strongly agree*). The four items were as follows: Being physically active is something … (1) I do without having to consciously remember; (2) I do without thinking; (3) I start doing before I realize I am doing it; (4) I do automatically. Scores across the four items were averaged to create a possible range of scores between 1 and 5. PA habit was assessed both at baseline and from then on, weekly over the course of the study. At baseline, the measure assessed participants' PA habit prior to the COVID‐19 pandemic (based on the month prior to the first lockdown). From then onwards, habit (measured via the Saturday diary) was assessed based on the most recent 7 days.

#### Physical activity frequency

When measured daily, participants reported whether they had done any vigorous activity, moderate activity, or walking for at least 10 min in duration. When measured weekly, participants reported the number of days on which they had engaged in vigorous activity, moderate activity, or walking of at least 10 min duration. Reports were used to calculate the total number of days each participant had engaged in moderate and/or vigorous PA and in walking over the past 7 days.

### Analysis

The hypotheses were tested using multi‐level modelling with PROC MIXED in SAS software, version 9.4. Multi‐level modelling allows assessment of longitudinal data, with observations over time nested within participants, with better capability than ANOVA‐based analyses at handling missing data, by utilizing multiple imputation versus listwise deletion of participants who have any missing data (Sterne et al., 2009). Hypothesis 1 was tested separately with PA habit strength and PA frequency as outcomes. Participants' reports of positive and negative intrinsic rewards from PA at each timepoint were used to predict these outcomes over 8 weeks post‐initial lockdown. To support the hypothesis, positive and negative intrinsic rewards were required to have significant main effects, and/or interactions with time, in predicting the outcomes, with the fixed effect estimates for negative intrinsic rewards expected to be greater than for positive intrinsic rewards.

Hypothesis 2 was tested using the same multi‐level models as for Hypothesis 1, but with negative intrinsic rewards and anxiety as the main factors, along with the interactions between anxiety and negative intrinsic rewards, and between anxiety, negative intrinsic rewards, and time. To support the hypothesis, we would expect to find a significant interaction between anxiety and negative intrinsic rewards or between anxiety, negative intrinsic rewards, and time in predicting the outcomes.

## RESULTS

There were only 18 total observations where there was a univariate outlier on any of the variables used in the current analyses, and all of these were outliers on positive intrinsic rewards. These were left in the dataset, because positive intrinsic rewards ranged only from 1 to 5; the outliers were merely low on the scale, compared to the mean of 4.04 (*SD* = 0.72) and were feasible responses.

There were missing observations throughout the eight observed weeks in the present analysis. Of the 580 individuals who consented to participate, there was a range from 252 (for Week 7) to 374 (Week 1) responses to the weekly surveys. Multi‐level modelling accounts for missing data in analyses so cases with missing data were not excluded listwise (from any analyses), and the software package SAS 9.4 utilized multiple imputation methods.

Descriptive information for all study variables for each week of the study is provided in Table 1.

**TABLE 1:** Descriptive information (means, SD) for study variables for each of 8 weeks of the study.

Week	Positive intrinsic rewards	Negative intrinsic rewards	Habit strength	Days walking	Days MVPA	Anxiety
**1**	4.05 (0.75)	3.93 (0.97)	2.97 (0.99)	4.11 (2.17)	4.85 (3.06)	2.82 (3.29)
**2**	4.08 (0.74)	3.81 (0.97)	2.99 (1.00)	4.12 (2.17)	4.78 (3.19)	2.83 (3.29)
**3**	4.04 (0.68)	3.80 (0.94)	3.01 (1.05)	4.40 (2.20)	5.41 (3.26)	2.59 (3.16)
**4**	4.05 (0.68)	3.84 (0.93)	2.99 (1.03)	4.33 (2.23)	5.20 (3.13)	2.72 (3.25)
**5**	4.04 (0.70)	3.80 (0.97)	2.96 (1.03)	4.42 (2.08)	5.19 (3.13)	2.65 (3.32)
**6**	4.02 (0.77)	3.77 (1.04)	2.98 (1.02)	4.42 (2.10)	5.39 (3.32)	2.34 (3.11)
**7**	4.06 (0.72)	3.77 (1.09)	3.01 (1.03)	4.35 (2.10)	4.85 (2.94)	2.36 (3.06)
**8**	4.02 (0.74)	3.74 (1.06)	2.95 (1.01)	4.29 (2.08)	4.96 (3.12)	2.24 (3.11)

*Note*: positive intrinsic rewards, negative intrinsic rewards, habit strength have possible values from 1 to 5. Anxiety scores could range between 0 and 12. Days Walking and Days MVPA (moderate or vigorous physical activity) could range from 0 to 7 days in each cell (for each week).

### Hypothesis 1

**FIGURE 1:**
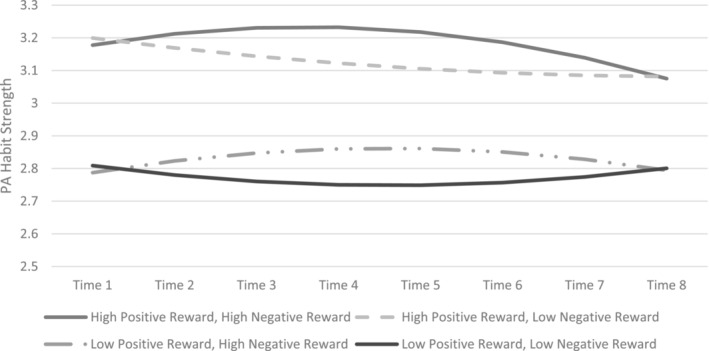
Physical activity habit strength during COVID‐19 restrictions, split by high versus low reports of positive intrinsic rewards (e.g., enjoyment; determined by 1 SD above or below the mean level of positive intrinsic rewards) and high versus low reports of negative intrinsic rewards (e.g., stress reduction; determined by 1 SD above or below the mean level of negative intrinsic rewards). Time is in weeks from baseline, which started 2 weeks after the beginning of the first lockdown in the UK. PA habit strength has a possible range of 1 to 5. The Y‐axis is truncated to better illustrate the statistically significant interactions of time (linear trend) and time‐squared (quadratic trend) with negative intrinsic rewards in predicting PA habit strength.

Fixed effects for PA habit strength as the outcome were significant for positive intrinsic rewards (estimate = 0.27, *SE* = 0.06, *t*[2176] = 4.46, *p* < 0.001) but not for negative intrinsic rewards (estimate = −0.05, *SE* = 0.05, *t*[2176] = −1.20, *p* = 0.23). There was a significant interaction between time and negative intrinsic rewards (estimate = 0.05, *SE* = 0.02, *t*[2176] = 2.33, *p* = 0.02) and between time‐squared and negative intrinsic rewards (estimate = −0.01, *SE* = 0.002, *t*[2176] = −2.48, *p* = 0.01). To interpret these results, we graphed the predicted values for habit strength over time, utilizing values for positive intrinsic rewards and negative intrinsic rewards at +/−1 SD from the mean values (see Figure 1).

Fixed effects for days of moderate or vigorous PA as the outcome was nonsignificant for positive and negative intrinsic rewards and for the interactions between these variables and time and time squared. Only time and time‐squared had significant main effects (respectively, estimate = 0.19, *SE* = 0.08, *t*(1863) = 2.42, *p* = 0.016; and estimate = −0.02, *SE* = 0.008, *t*(1863) = −3.02, *p* = 0.003), indicating the number of days with moderate or vigorous activity increased over time but at a decreasing rate.

Similarly, fixed effects for days of walking as the outcome were nonsignificant for positive and negative intrinsic rewards, and the interactions between these variables and time and time squared. Only time and time‐squared had significant main effects (respectively, estimate = 0.14, *SE* = 0.06, *t*(1994) = 2.42, *p* = 0.016; and estimate = −0.01, *SE* = 0.006, *t*(1994) = −2.36, *p* = 0.018), indicating the number of days of walking increased over time but at a decreasing rate.

### Hypothesis 2

**FIGURE 2:**
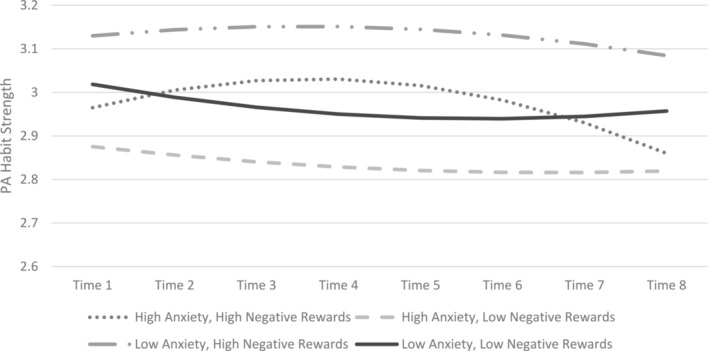
Physical activity habit strength during COVID‐19 restrictions on movement, split by high versus low anxiety (determined by 1 SD above or below the mean level of anxiety) and high versus low reports of negative intrinsic rewards (e.g., stress reduction; determined by 1 SD above or below the mean level of negative intrinsic rewards). Time is in weeks from baseline, which started 2 weeks after the beginning of the first lockdown in the UK. PA habit strength has a possible range of 1 to 5. The Y‐axis is truncated to better illustrate the statistically significant interactions of time (linear trend) and time‐squared (quadratic trend) with anxiety in predicting PA habit strength.

The results for Hypothesis 2 are similar to the results of Hypothesis 1. For habit strength as the outcome, the interactions between negative intrinsic rewards and time (estimate = 0.04, *SE* = 0.02, *t*[2192] = 1.98, *p* = 0.048), and between negative intrinsic rewards and time‐squared, were significant (estimate = −0.005, *SE* = 0.002, *t*[2192] = −2.17, *p* = 0.03). There was a main effect of anxiety on habit strength (estimate = −0.03, *SE* = 0.013, *t*[2192] = −2.17, *p* = 0.03), but the expected interactions between anxiety and negative intrinsic rewards (estimate = −0.004, *SE* = 0.01, *t*[2192] = −0.29, *p* = 0.77) and between anxiety, negative intrinsic rewards, and time (estimate = 0.002, *SE* = 0.006, *t*[2192] = 0.36, *p* = 0.72) were not significant (Figure 2).

Also, similarly to the results for Hypothesis 1, the results for days of moderate and vigorous activity and for days of walking had only significant fixed effects for time and time‐squared, with the outcomes increasing over time at a progressively slower rate. Data file, syntax, and full results are available for all of the above analyses on the open science framework (https://osf.io/ra4sw/?view_only=8b730dcd6ab841119943b7cfaae7c40a).

## DISCUSSION

The current study evaluated whether individuals' reports of positive and negative intrinsic rewards from engaging in PA would be associated with their PA habit strength and frequency during a period of 8 weeks at the start of the COVID‐19 pandemic. We hypothesized that, given the rare globally shared context of a substantial stressor, in which individual levels of anxiety may be particularly high, negative intrinsic rewards would be more strongly related to PA habit strength and frequency than would be positive intrinsic rewards. Further, we hypothesized that this relationship between negative intrinsic rewards and outcomes would be particularly strong for those reporting higher levels of anxiety. While we did not expect the underlining mechanisms of positive and negative intrinsic reward to differ, we did expect there to be differing effects from these two types of rewards. More specifically, in stressful times and/or when sufficient time is perceived to be lacking, people are more likely to stick to engaging in behaviors that they deem essential (Epel et al., 2018). As such, during the COVID‐19 pandemic, a time of high stress, it was expected that PA engagement would be stronger for those who have negative intrinsic rewards for PA as they would deem the engagement in PA as being more essential as they are not just doing it for enjoyment, but for reduction of negative emotions.

Our hypotheses were generally not supported. PA habit strength was most strongly related to positive intrinsic rewards versus negative intrinsic rewards. Further, although anxiety had a significant negative main effect on PA habit strength and PA frequency, there was no interaction with negative intrinsic rewards, meaning individuals high in anxiety were less physically active and had weaker PA habits even if they reported experiencing stress reduction and better mood through engaging in PA. Although this fits with the literature that shows anxiety and stress interfere with engagement in PA (Burg et al., 2017; Wanjau et al., 2023), it does not seem to fit with the literature suggesting that negative intrinsic rewards (stress reduction and mood improvement) act as a motivator for PA and are associated with greater overall engagement in PA (Ednie & Stibor, 2017; Stults‐Kolehmainen & Sinha, 2014). These findings could be explained by the study being conducted during the COVID‐19 pandemic. Though the pandemic provided the opportunity to better compare how negative and positive intrinsic rewards impact PA habit and frequency during times of high stress, the pandemic was a novel stressor, meaning the types of negative emotions elicited from the pandemic could be unique and different from more common high stress time periods (Li et al., 2024). However, given that both pandemic‐like stressors and other novel stressors are likely to occur with more frequency as the population continues to grow and resources continue to be depleted (Suk et al., 2020), the results of this study can be applied to future stressful world events.

Most of the past research that utilizes the SDT to explore the beneficial effects of intrinsic rewards and intrinsic motivation has neglected to explore the different types of intrinsic reward. The present study addressed this by assessing how the different intrinsic reward types attained through PA impact PA habits during times of high stress. Results indicate that the development of positive emotions through PA engagement (i.e., positive intrinsic reward, such as enjoyment) was associated with greater PA habits, but the removal or mitigation of negative emotions through PA engagement (i.e., negative intrinsic reward, such as experiencing reduced stress/anxiety) was not. These results suggest that future intervention efforts may optimally focus on fostering the attainment of joy, pleasure, and satisfaction from PA engagement rather than focusing on ways to increase stress reduction through PA. These results are supported by past literature including a systematic review, which found that reports of positive affect during PA engagement had a significant, positive relationship to future PA behaviors (Rhodes & Kates, 2015). Notably, however, this same review also indicated that there was no significant impact of post PA effect on future PA behaviors. These results combined with the results of the present study indicate the need to enjoy the act of engaging in PA for a PA behavior to be sustained across time, and that the attainment of positive affective emotions post activity or the removal negative affective emotions through activity are less import factors in the promotion of PA maintenance. Thus, future interventions may best concentrate on promoting positive intrinsic rewards during the actual PA process rather than post PA intrinsic rewards.

The results were surprising, given the widely acknowledged barrier to exercise that stress and anxiety present to individuals (Ednie & Stibor, 2017) and that under stress, individuals with exercise habits increase their activity levels (Stults‐Kolehmainen & Sinha, 2014). Additionally, the significant interactions between time and negative intrinsic rewards and between time‐squared and negative intrinsic rewards, as depicted in Figures 1 and 2 for tests of both hypotheses, were unexpected and difficult to interpret for meaningful clinical implications. The unexpected results may be due to the limitations of the data, including the timing of the assessments being untied to exercise events and the correlational nature of the data. Specifically, it is possible that the general positive affect experienced after an exercise session from either positive or intrinsic negative rewards was most salient when participants answered the surveys (which were retrospective), and participants may have undervalued and underreported the importance of stress reduction or reduction in other negative pre‐exercise states for engaging in exercise. Other experience sampling methods, such as surveys that assess stress levels before and after exercise along with enjoyment of an exercise activity (during and after) could be utilized to better evaluate the influence of stress and stress reduction from exercise on subsequent exercise habit formation and maintenance. Further, although it would not be possible to randomly assign individuals to be motivated by positive versus negative intrinsic rewards from exercise or to study such a large sample in a lab setting during a pandemic or other real‐world event, future research could take advantage of experimental methods to study the relative advantages of strategies that aim to help individuals experience negative versus positive intrinsic rewards from exercise.

Other limitations of the present study include that the sample was fairly homogenous, with a majority identifying as White, female, and in a younger age range, limiting generalization to other populations. Further, all measures were self‐reported and may have been affected by social desirability bias or poor recall. There were also many missing observations over time, which is not unusual for longitudinal survey studies and is well‐handled by multi‐level modelling when missing completely at random. However, the missing data could have influenced the results, for example, individuals may have avoided completing surveys when they were inactive or under particularly high stress or anxiety. Additionally, the results of the present study were just a snapshot in time and were limited to self‐report data. Intrinsic behavioral rewards may play differing roles at different stages of PA development (i.e., initiation, maintenance, and habituation); as such, the data included individuals who may have been in any of these stages of PA adoption, complicating the relationships between experience of intrinsic rewards and habit strength over time. For example, for those forming a PA habit during the period of the study, the time may not have been sufficiently long to observe substantial change in habit strength.

Overall, the results suggest that PA habit and frequency are highest when positive intrinsic rewards (e.g., enjoyment) are high, even in times of particularly high stress. These results suggest that interventions might best focus on fostering positive intrinsic rewards to engage in PA. However, future research should continue to explore the role of negative intrinsic rewards in PA habit formation through methods that can capture affective and stress states prior to and after exercise and/or experimental methods that compare the short‐ and long‐term effectiveness of interventions designed to help participants realize the negative intrinsic rewards of exercise versus positive intrinsic rewards from exercise.

## CONFLICT OF INTEREST STATEMENT

The authors have no competing interests to declare.

## ETHICS STATEMENT

All participants provided informed consent prior to data collection. Ethical approval was granted by the University of Hertfordshire's Health Science Technology and Engineering Ethics Committee with Delegated Authority (ECDA; Protocol number: LMS/SF/UH/04136.

## Data Availability

Data are available from the corresponding author for verification purposes. SAS data file, syntax, and output are available on the open science framework at https://osf.io/ra4sw/?view_only=8b730dcd6ab841119943b7cfaae7c40a.
